# Epicardially Placed Bioengineered Cardiomyocyte Xenograft in Immune-Competent Rat Model of Heart Failure

**DOI:** 10.1155/2021/9935679

**Published:** 2021-07-24

**Authors:** Ikeotunye Royal Chinyere, Pierce Bradley, Joshua Uhlorn, Joshua Eason, Saffie Mohran, Giuliana G. Repetti, Sherry Daugherty, Jen Watson Koevary, Steven Goldman, Jordan J. Lancaster

**Affiliations:** ^1^Sarver Heart Center, University of Arizona, Tucson, AZ, USA; ^2^Physiological Sciences GIDP, University of Arizona, Tucson, AZ, USA; ^3^Department of Biomedical Engineering, University of Arizona, Tucson, AZ, USA

## Abstract

**Background:**

Human induced pluripotent stem cell-derived cardiomyocytes (hiPSC-CMs) are under preclinical investigation as a cell-based therapy for heart failure post-myocardial infarction. In a previous study, tissue-engineered cardiac grafts were found to improve hosts' cardiac electrical and mechanical functions. However, the durability of effect, immune response, and *in vitro* properties of the tissue graft remained uncharacterized. This present study is aimed at confirming the graft therapeutic efficacy in an immune-competent chronic heart failure (CHF) model and providing evaluation of the *in vitro* properties of the tissue graft.

**Methods:**

hiPSC-CMs and human dermal fibroblasts were cultured into a synthetic bioabsorbable scaffold. The engineered grafts underwent epicardial implantation in infarcted immune-competent male Sprague-Dawley rats. Plasma samples were collected throughout the study to quantify antibody titers. At the study endpoint, all cohorts underwent echocardiographic, hemodynamic, electrophysiologic, and histopathologic assessments.

**Results:**

The epicardially placed tissue graft therapy improved (*p* < 0.05) *in vivo* and *ex vivo* cardiac function compared to the untreated CHF cohort. Total IgM and IgG increased for both the untreated and graft-treated CHF cohorts. An immune response to the grafts was detected after seven days in graft-treated CHF rats only. *In vitro*, engineered grafts exhibited responsiveness to beta-adrenergic receptor agonism/antagonism and SERCA inhibition and elicited complex molecular profiles.

**Conclusions:**

This hiPSC-CM-derived cardiac graft improved systolic and diastolic cardiac function in immune-competent CHF rats. The improvements were detectable at seven weeks post-graft implantation despite an antibody response beginning at week one and peaking at week three. This suggests that non-integrating cell-based therapy delivered by a bioengineered tissue graft for ischemic cardiomyopathy is a viable treatment option.

## 1. Introduction

Heart failure (HF) with reduced ejection fraction carries a mortality of over 30% within five years of the initial hospitalization [1, 2]. While management of the HF patients continues to improve, there remains a need for new therapeutic options that directly mitigate disease progression. The development of human induced pluripotent stem cells (hiPSCs) provides an opportunity to utilize novel cell types in engineered tissue platforms for regenerative therapy [3, 4]. For the first time, hiPSC-derived cardiomyocytes (hiPSC-CMs) are being evaluated in HF patients at Osaka University, where cardiothoracic surgeons are implanting hiPSC-CM sheets on the epicardium (NCT04696328).

A previous study from our laboratory demonstrated that tissue-engineered (TE) cardiac grafts composed of human neonatal dermal fibroblasts seeded on bioabsorbable scaffolds induced angiogenesis and increased myocardial blood flow in rodents with HF after myocardial infarction (MI) [5]. In addition, a more recent study showed that hiPSC-CMs seeded on the same fibroblast scaffold improved cardiac function in the same rodent model of HF [6] Interestingly, these benefits were observed using a xenograft transplant of human fibroblasts and hiPSC-CMs in immune-competent Sprague-Dawley rats, in which we have shown that the transplanted cells do not persist [6].

The goals of this study were to (1) confirm the reproducibility of a previous hiPSC-CM TE graft study [6], (2) test the durability of effect in a longer duration study, (3) provide preliminary characterization of the humoral immune response following graft therapy in this immune-competent xenograft model, and (4) perform a comprehensive *in vitro* electrophysiologic and mechanical characterization of the TE graft both *in vitro* and *in vivo*. HiPSC-CMs were utilized in this study rather than rodent or murine iPSC-CMs because this study was designed as a preclinical evaluation of a novel therapeutic that is being optimized for clinical implementation. We hypothesized that implanting these TE cardiac grafts onto the epicardium of immune-competent rats in HF would result in improvements in cardiac function and that durability would persist despite the transient nature of the implant.

## 2. Materials and Methods

### 2.1. Myocardial Infarction, Chronic Heart Failure, and Graft Deployment

Three weeks after permanent left coronary ligation, chronic heart failure (CHF) rats were randomly assigned to either TE graft treatment or untreated CHF for seven weeks. Rats assigned to untreated CHF underwent the second surgical approach without graft treatment. Prior to surgical implantation, each graft was removed from the incubator and transferred to a 35-millimeter dish and rinsed in 1x phosphate-buffered saline. The chest was reopened via partial left thoracotomy, and one graft was implanted over the infarcted area, bridging healthy to healthy myocardium. The graft was secured using 3-4 simple interrupted suture stitches for the periphery of the graft and a single simple stitch for the center of the graft.

### 2.2. Ex Vivo Diastolic Pressure-Volume Relationships

Three diastolic pressure-volume (*P*/*V*) relationship curves were obtained within ten minutes of induced cardiac arrest and analyzed for dead volume, stiffness constants, left ventricular (LV) pressure, and standardized volume analyses [7].

### 2.3. Histopathologic Assessment

For rodent hearts that did not undergo *ex vivoP*/*V* analysis, the right ventricles were excised and the LV specimens were fixed in 10% paraformaldehyde, prior to paraffin embedding and cutting into three short-axis sections. Myocardial sections were stained using Gomori's Trichrome to characterize the three-dimensional distribution of healthy myocardium and scar tissue [8].

### 2.4. Immune Response

To characterize the humoral response to the engineered graft, plasma was isolated serially from all rats. Whole blood was collected from the tail vein at baseline prior to infarction, three weeks following infarction immediately prior to graft implantation (week 0) and at weeks 1, 3, and 7 post-graft implantation.

Plasma was analyzed to quantify total nonspecific IgM and total nonspecific IgG using sandwich enzyme-linked immunosorbent assay (ELISA; Thermo Companies, Denver, CO, USA). To assess the graft-specific IgG response, serially collected plasma samples were tested by flow cytometry. In brief, plasma from CHF rats and graft-treated rats was decomplemented for thirty minutes in a 56°C water bath prior to incubation with hiPSCs-CMs or fibroblasts (viability confirmed using Zombie Violet) at a ratio of 1 *μ*L of plasma to one million cells in a 100 *μ*L suspension. The presence of rat anti-fibroblast primary antibodies and rat anti-hiPSC-CM primary antibodies was confirmed using conjugated secondary mouse anti-rat IgM and goat anti-rat IgG antibodies, respectively, each linked to a fluorophore (BioLegend, San Diego, CA, USA).

### 2.5. In Vitro Graft Assessment

The TE graft described in this study is composed of a cellularized bioabsorbable polyglactin 910 knitted mesh. The meshes are cut into 1.6 cm diameter circular disks. The 1.6 cm disks are placed into the base of a 24-well plate and inoculated with human neonatal dermal fibroblasts and hiPSC-CMs in a ratio between 1 : 1 and 1 : 2, respectively. The hiPSC-CMs are terminally differentiated and express >95% cTnT and display contractions 24 hrs after integration into the grafts. The grafts can be manufactured easily in large batches. The grafts adhere tightly to the heart and are secured in place with 2-3 spot sutures.

The TE grafts were assessed for intracellular calcium concentration flux and mechanical contraction at increasing rates of depolarization (1 hertz/60 beats per minute to 3 hertz/180 beats per minute) with the CellOPTIQ® platform. Intracellular calcium was quantified optically using *Fura 4* florescent indicator, and mechanical displacement was quantified using MuscleMotion image analysis software [9]. Thapsigargin, an irreversible sarcoendoplasmic reticulum ATPase (SERCA) isoform 2a inhibitor, was utilized to assess the contribution of the sarcoplasmic reticulum to the overall intracellular calcium flux. The detailed methods for these electrophysiologic (EP) assays have been previously described [10].

## 3. Results

### 3.1. Myocardial Infarction, Chronic Heart Failure, and Graft Deployment

**Figure 1:**
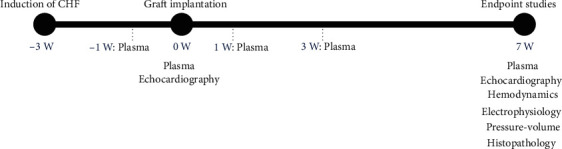
Timeline of rat surgeries. Chronologic study timeline in weeks (W). Sham controls received a thoracotomy at the time of CHF induction but did not undergo permanent coronary occlusion. A portion of CHF control rats were randomly selected to receive the graft therapy. CHF control rats that were not selected for graft therapy still received a sham lateral thoracotomy. Plasma was obtained by removing the superior-most portion of the centrifuged whole blood for antibodies.

A study timeline for the *in vivo* procedures has been provided for reference (Figure 1). Each graft implantation was considered successful based on visual assessment at the time of chest closure; the graft was easily handled and effectively implanted into each recipient rat covering the site of injury (Supplemental Figure 1). During the study, one sham rat died within 48 hours of the thoracotomy surgery; the remaining sham rats survived to the completion of the study. CHF rats exhibited the anticipated survival rate of 60%; at the second thoracotomy, three CHF rats died. Two graft-treated CHF rats died shortly after the second thoracotomy surgery for graft implantation.

^∗^The remainder of *in vivo* results (Supplemental Figure 2 and Supplemental Figure 3) can be found in the manuscript supplement.

### 3.2. Ex Vivo Diastolic Pressure-Volume Relationships

**Figure 2:**
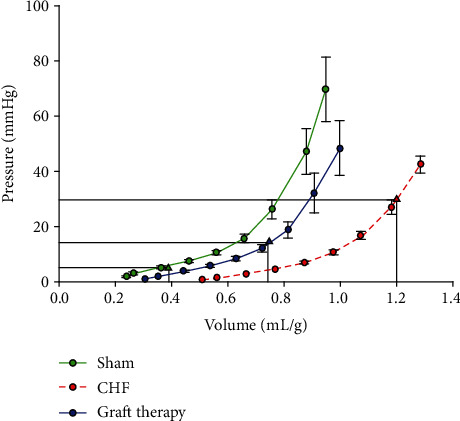
Rat diastolic pressure-volume relationship. *Ex vivo* pressure-volume (*P*/*V*) diastolic filling curves of sham (*n* = 4), CHF (*n* = 5), and graft-treated rats (*n* = 7). Left ventricular volumes were normalized to each rat's body weight. CHF results in a rightward shift in *P*/*V* filling curve and an increase in the operating volume. The graft-treated rats have a *P*/*V* filling curve shifted back towards sham and a lower operating volume, consistent with reversal of maladaptive remodeling related to compliance.

An exponential-shaped *P*/*V* curve was fit to the data for all three groups, sham (*n* = 4), CHF (*n* = 5), and graft-treated CHF (*n* = 7) (Figure 2). The *P*/*V* curve from the sham animals was displaced to the left toward the pressure axis, and the CHF curve displaced to the right, as expected with maladaptive LV remodeling. The TE graft-treated rat hearts had a *P*/*V* curve that resided between the sham and CHF groups, a shift toward normal indicating a partial reversal of LV remodeling.

### 3.3. Histopathologic Assessment

**Figure 3:**
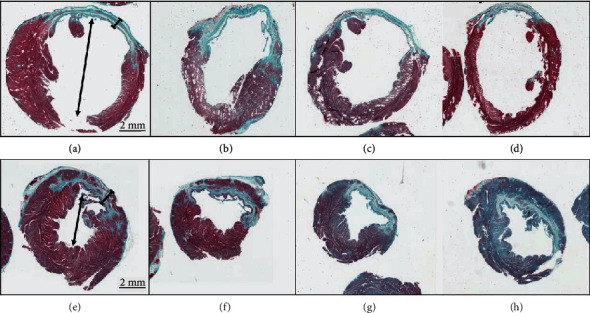
Cardiac histopathology. Representative left ventricular histopathology sections from 4 unique CHF rats (panels a–d) and 4 unique graft-treated rats (panels e–h). Masson's Trichrome stain was performed on 5 *μ*m transverse sections at the level of the papillary muscles. Micrographs (0.6x) have a 2 mm scale bar for reference. CHF rats exhibit large transmural scars in addition to dilated ventricular cavities, as anticipated with the adverse remodeling from heart failure. Graft-treated hearts exhibit increased wall thickness and increased myocyte density in the previously infarcted region. They also exhibit smaller ventricular cavities compared to the CHF controls.

All evaluated LV sections were from equal ventricular heights, ensuring appropriate comparison between short-axis sections. Randomly selected graft-treated CHF sections revealed smaller ventricular cavity diameters, increased ventricular anterior wall thickness, and increased anterior wall myocyte densities as compared to randomly selected untreated CHF controls (Figure 3).

### 3.4. Immune Response

**Figure 4:**
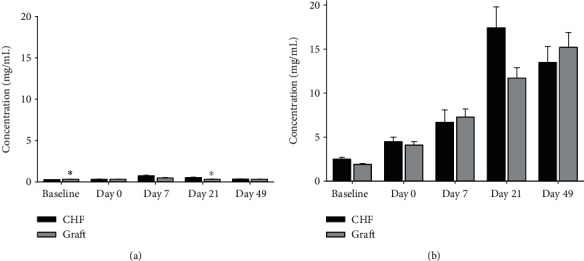
Rat immunologic titers. Total IgM (a) and IgG (b) values were evaluated in CHF (*n* = 5-10; black bars) rats and graft-treated rats (*n* = 5; gray bars) at baseline (prior to infarction), day 0 (immediately prior to graft therapy), and days 7, 21, and 49 post-graft therapy. Serum IgM and IgG values for the graft-treated rats were predominately equal to the values for CHF controls with the exception of IgM at baseline and at day 21. Values are mean ± standard error of the mean. ^∗^Unpaired *t*-test versus CHF, *p* < 0.05.

Total IgM and IgG antibody response was quantified using ELISAs (Figure 4). There were differences between the amount of IgM in CHF rats (*n* = 10) and graft-treated CHF rats (*n* = 8) at baseline (0.33 ± 0.01 versus 0.29 ± 0.01 mg/mL, *p* = 0.0073) and between CHF rats (*n* = 5) and graft-treated rats (*n* = 8) at 21 days post-graft therapy (0.34 ± 0.01 versus 0.55 ± 0.08 mg/mL, *p* = 0.0462). No significant IgM differences were observed at all other time points. There were no differences in the level of detectable IgG between CHF and graft-treated CHF rats.

**Figure 5:**
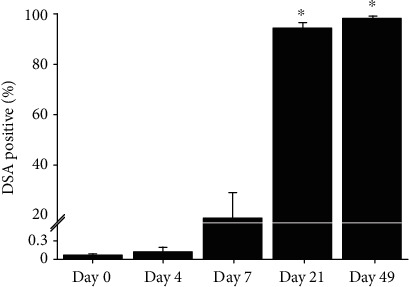
Rat-specific immune response: graft therapy. The graft-specific IgG antibody (donor-specific antibody (DSA)) response was evaluated in graft-treated rat serum (*n* = 7-8) at days 0, 4, 7, 21, and 49 post-graft implantation. A subphysiologic amount of DSA was detected on hiPSC-CMs and fibroblasts at days 0 and 4, with a stepwise increase between days 7 and 49 (1 *μ*L plasma:100 *μ*L cellular suspension). Despite the presence of these graft-specific antibodies, reversal of maladaptive remodeling was observed in graft-treated rats. ^∗^Unpaired *t*-test versus day 7, *p* < 0.05.

Beginning from seven days post-graft implantation, graft-specific IgG antibodies against the fibroblasts and hiPSC-CMs (Figure 5) were detected in the plasma collected from graft-treated CHF rats (*n* = 7-8, 58.7 ± 11.8% positive hiPSC-CMs). These antibodies were not detected in the plasma of the untreated CHF group. Beyond seven days post-graft implantation, the graft-specific antibodies steadily increased over the duration of the study up to the study endpoint and at seven weeks post-graft implantation (58.7 ± 11.8% to 99.3 ± 0.1% positive cells, *p* = 0.0069) (Figure 5). No graft-specific antibodies were detected in the plasma of the untreated CHF group at any time point.

### 3.5. In Vitro Graft Assessment

Five days after the initiation of coculture, engineered grafts contracted spontaneously and synchronously at 71 ± 7 beats-per-minute during culture (Supplemental Video 1). Contraction was defined as equal displacement of left and right edges of the graft towards the center of the graft.

**Figure 6:**
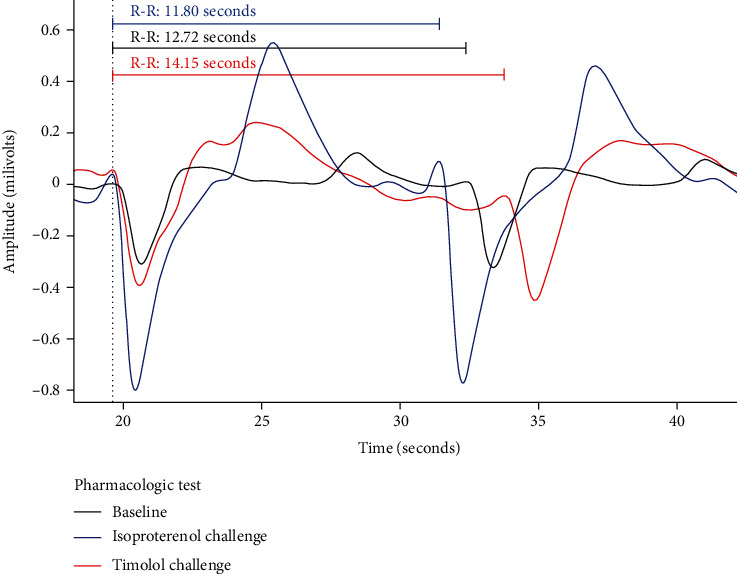
Unipolar electrograms from engineered graft. Superimposed colorized tracings depicting unipolar voltage electrograms (UVE) acquired utilizing a multielectrode array on one engineered cardiac graft. The black tracing exhibits the control depolarization (QS wave) and repolarization (T wave) waveforms via UVE collected without any pharmacologic manipulation. The blue tracing exhibits a UVE obtained from the same engineered graft exposed to isoproterenol (1*E*-2 M); a physiologic shortening of the QS-QS interval and QT interval is observed. The red tracing exhibits a UVE obtained from the same engineered graft exposed to timolol (1*E*-4 M); a physiologic lengthening of the QS-QS interval and QT interval is observed.

A commercial multielectrode array (NeuroNexus Technology Ann Arbor, MI, USA) was used to depict unipolar voltage electrograms, which revealed clear depolarization and repolarization waveforms (Figure 6). The large field-of-view waveforms produced a baseline QS wave representing depolarization of the engineered cardiac graft and a subsequent T wave representing repolarization. Each graft displayed synchronous electrical activity as measured by a multielectrode array and exhibited coordinated mechanical displacement.

Pharmacologic challenge with isoproterenol (*n* = 8) revealed a physiologic shortening of the R-R interval relative to baseline (*n* = 8) (99.8 ± 0.4 milliseconds (ms) versus 106.9 ± 0.4 ms, *p* < 0.0001). Furthermore, electrograms revealed a physiologic shortening of the QT interval (78.6 ± 1.04 ms versus 101.2 ± 1.50 ms, *p* < 0.0001) with isoproterenol (*n* = 6), relative to baseline (*n* = 3). Changes in the electrogram amplitude between electrogram waveforms is likely due to differences in adhesion forces between the hydrophobic array and the engineered cardiac graft from differing amounts of culture media in the petri dish (Figure 6). After thorough washing and a return to preisoproterenol electrogram characteristics, pharmacologic challenge with timolol (*n* = 4) revealed a physiologic prolongation of the QS-QS interval (147.7 ± 1.5 ms versus 106.9 ± 0.4 ms, *p* = 0.0234) as well as a physiologic prolongation of the QT interval (116.5 ± 3.88 ms versus 101.2 ± 1.50 ms, *p* < 0.0001).

Additional electrophysiologic evaluation of the TE grafts with the Maestro MEA system revealed sinus-like regular field potentials with negative or biphasic QRS complexes and clearly discernable T waves in nine independent grafts, each in its own well (Supplemental Figure 4). No P waves were observed in the field potentials. Contact between the MEA plate and the engineered cardiac graft was optimal even during media changes. Adrenergic stimulation and inhibition revealed the expected physiologic responses with graded increases in beat rate (R-R interval) and graded decreases in QT interval after exposure to isoproterenol and graded decreases in beat rate and graded increases in QT interval after exposure to sotalol.

**Figure 7:**
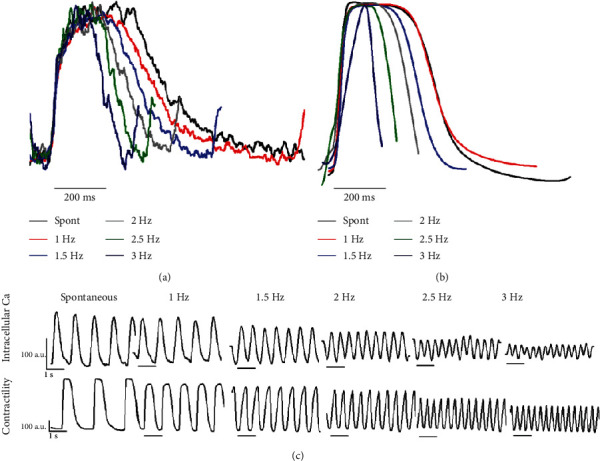
Intracellular calcium flux and mechanical contraction. Superimposed colorized tracings depicting the (a) height-normalized intracellular calcium flux waveforms from hiPSC-CMs and fibroblasts integrated into an engineered cardiac graft and the (b) height-normalized mechanical contraction waveforms from the same graft in milliseconds (ms). In black, the graft is beating at its spontaneous (spont) rate. The graft was then paced with at incrementally increasing rates between one Hertz (1 Hz) and three Hertz (3 Hz) to characterize the engineered cardiac graft's flux-frequency and force-frequency relationships. (c) The original intracellular calcium and mechanical contraction tracings, with arbitrary units on the *y*-axis and time in seconds on the *x*-axis. A decreasing calcium-flux and contraction amplitude is observed with increasing pacing frequency (negative flux-frequency and force-frequency relationships), suggesting functional deficit in the contractile machinery in this engineered cardiac graft.

Intracellular calcium flux and contraction force assessment as the spontaneous intrinsic beat rate and pacing up to 3 hertz revealed pacing-induced changes which suggested an immature sarcoplasmic reticular contribution to calcium flux and subsequent contraction force (Figure 7). The engineered graft revealed a negative flux-frequency relationship and a negative force-frequency relationship with respect to intracellular calcium flux and contraction force.

Interestingly, comparison of calcium flux rise time and duration to 50% versus cycle length in the engineered grafts revealed decreased calcium flux rise time with thapsigargin exposure, but no change in calcium influx waveform duration to 50% (Supplemental Figure 5).

### 3.6. Induced Pluripotent Stem Cell-Derived Cardiomyocytes

**Figure 8:**
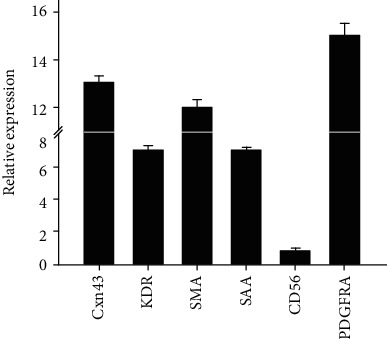
Molecular phenotype of engineered cardiac grafts. Quantitative polymer chain reaction was utilized to assess the molecular phenotype of fully constituted, implantation-ready engineered cardiac grafts (*n* = 2). The phenotype is defined by expression of connexin 43 (Cxn43), kinase insert domain receptor (KDR), smooth muscle actin (SMA), sarcomeric alpha actinin (SAA), CD56, and platelet-derived growth factor receptor-*α* (PDGFRA). Grafts were composed of hiPSC-CMs and human fibroblasts on a bioabsorbable construct. Data are reported relative to a fibroblast-only cardiac graft as a control.

Tissue grafts generated with hiPSC-CMs (*n* = 2) expressed increased levels of connexin 43 (*Cxn43*), kinase insert domain receptor (*KDR*), smooth muscle actin (*SMA*), sarcomeric alpha actinin (*SAA*), cluster of differentiation 56 (*CD56*), and platelet-derived growth factor receptor-*α* (*PDGFRA*) compared to a fibroblast-only graft that did not contain hiPSC-CMs (Figure 8).

## 4. Discussion

The goals of this study were to (1) confirm the reproducibility of a previous hiPSC-CM TE graft study [6], (2) test the durability of effect in a longer duration study, (3) provide preliminary characterization of the humoral immune response following TE graft therapy in this immune-competent xenograft model, and (4) perform a comprehensive *in vitro* electrophysiologic and mechanical characterization of the TE graft *in vitro* prior to implantation. We hypothesized that implanting the TE cardiac grafts onto the epicardium of immune-competent rats in HF would result in improvements in cardiac function and that durability would persist despite the transient nature of the implant.

Previously, we reported that an engineered cardiac xenograft composed of human neonatal fibroblasts and hiPSC-CMs altered adverse LV remodeling post-MI [6]. These studies were conducted in immune-competent Sprague-Dawley rats. As confirmed by quantitative polymerase chain reaction and immunohistochemistry, no transplanted cells persisted beyond 21 days post-graft implantation, yet this graft resulted in functional benefit [6]. In the present study, that same breed of rat, myocardial injury, and surgical approaches were used (Supplemental Figure 1). However, the *in vivo* timeline was increased from three weeks to seven weeks (Figure 1). Consistent with our previous reports, functional benefit persisted to the later time point demonstrating durability of response. The observed decrease in LV end-diastolic pressure (EDP) and relaxation constant Tau and the increased ±*dP*/*dt* and peak-developed pressure showed improved LV hemodynamics post-MI in the context of comparable afterload (Supplemental Figure 2).

The *ex vivoP*/*V* analysis (Figure 2) demonstrated a beneficial shift in the LV diastolic *P*/*V* relationship towards the pressure axis with a decrease in the operating LV end-diastolic volume. The decrease in the EDP with treatment (Supplemental Figure 2) coupled with the decrease in operating LV end-diastolic volume means that the TE graft did not create any restrictive cardiac physiology, or the LV pressures and volumes would have increased. These findings suggest that the graft therapy mitigates maladaptive LV remodeling, reinforcing the *in vivo* invasive hemodynamic findings and reproducing the previously reported positive findings after three weeks with epicardial graft therapy [6]. Although an *in vivo* improvement in LV ejection fraction (EF) was not observed (Supplemental Figure 3), the shortcomings of using EF as an index of LV contractile function have been well defined [11].

Increased LV anterior wall thickness was confirmed by histopathology (Figure 3) and echocardiography (Supplemental Figure 3) as compared to CHF control. A notable increase in abundance of the viable myocardium was observed in the previously infarcted heart of graft-treated animals through Gomori's Trichrome staining. Consistent with our previous findings, these myocytes were not from the xenograft implant. We hypothesize that the synergistic effects of angiogenesis, paracrine signaling, and electrical stabilization mediated by the xenograft therapy facilitate retention of stunned/hibernating myocardium.

Molecular assessment of the engineered graft revealed a molecular profile consisting of upregulated endothelial, fibroblast, and cardiomyocyte markers (Figure 8). This finding suggests a paracrine-mediated enhancement of the of cell activity during culture *in vitro*. These data build on previously published data showing the presence of multiple growth factors and cytokines released from the engineered tissue grafts. Mechanistically, these findings are similar to those reported with mesenchymal precursor cells [12], wherein the cells do not persist for any appreciable time and soluble factors such as SDF-1, VEGF, and ANG-1 in addition to immune macrophage activity have been shown to enhance native cardiomyocyte survival and contribute to neovascularization [12]. We do speculate about the broad epicardial administration of graft and potential roles the epicardium may play given that the epicardium has been recently described as a hub for heart regeneration [13, 14].

Clinically, the reported hemodynamic changes are similar to the benefits of angiotensin-converting enzyme inhibition (ACE-I) in the treatment of HF as described by Pfeffer et al. in 1985 [15]. Specifically, their *P*/*V* analyses, similar to ours in Figure 2, showed partial reversal of maladaptive LV remodeling with ACE-I in HF. Their preclinical findings served as the basis for the use of ACE-I as guideline-directed medical therapy for HF patients, which eventually was shown to decrease mortality in these patients. Our hemodynamic data demonstrate a similar magnitude response to their data, indicating that this hiPSC-CM graft therapy could have an important therapeutic benefit in treating HF. Specifically, the lower LV operating end-diastolic volume, shortened LV relaxation constant Tau, and smaller LV chamber size could improve HF patients' quality of life by decreasing shortness of breath and improving exercise tolerance as well as potentially a decrease HF patient mortality. Furthermore, these preclinical findings suggest that this graft therapy may facilitate both decreased HF morbidity and mortality by preserving ventricular systolic function in patients with HF as well as improved quality of life via minimal deterioration of diastolic function and cardiac exercise tolerance.

To better understand the impact of the immune-competent model, we conducted a preliminarily evaluation of the humoral immune response. Total IgM and IgG titers (Figure 4) showed equivalent antigen exposure between the untreated CHF rats and graft-treated CHF rats, meaning that exposure to the xenograft itself did not produce a significant increase in antibody titers. Furthermore, graft-treated CHF rats generated donor-specific IgG antibodies that were detectable at 7 days and peaked at 21 days post-implantation (Figure 5). These data demonstrate that despite the cells being detected and likely eliminated by the host's immune system, cardiac transplantation of xenogeneic cells is safe and can confer a functional benefit [16, 17]. Clinical extrapolation of these findings would suggest that despite immune recognition of this engineered graft therapy in HF patients, the immune recognition should not influence patient eligibility for a future heart transplant any more than receiving blood product transfusions or having multiple pregnancies prior to heart transplantation.

An *in vitro* electrophysiologic and mechanical characterization of the engineered graft was performed. Preliminary assessment of the engineered graft with light microscopy showed rapidly spontaneous and synchronous mechanical activity (Supplemental Video 1). *In vitro* pharmacologic challenges to the engineered graft showed that the hiPSC-CMs are capable of appropriate physiologic responses to beta-adrenergic receptor agonism and antagonism (Figure 6, Supplemental Figure 4). Further assessment of the hiPSC-CMs revealed negative calcium flux-frequency and force-frequency relationships (Figure 7). The sarcoplasmic reticular contribution to intracellular hiPSC-CM calcium rise time was significantly impacted with increasing cycle length (Supplemental Figure 5).

### 4.1. Limitations

An additional control group consisting of CHF rats that received hiPSC-CM-free grafts, that is only the polyglactin 910 knitted mesh and the human neonatal dermal fibroblasts, would have provided additional experimental clarity. However, a previously completed study described such a graft's effect as angiogenic without facilitating improvements in hemodynamic parameters [5].

In addition, no assay regarding the survival rate of implanted hiPSC-CMs was performed in this study. Nonetheless, our previous publication revealed that no xenografted hiPSC-CMs survived beyond 21 days post-implantation [6]. Due to these data, we conclude this graft is non-integrating.

Finally, one of the stated goals of this study was to provide a preliminary characterization of the humoral immune response following TE graft therapy. Though the goal was accomplished using total and graft-specific IgM and IgG titers, it is necessary to acknowledge that evaluation of the innate immune response in the target organ would have provided a more comprehensive perspective regarding transplanted cell fate and microenvironment quality. Regardless of the exact local innate response, the transplanted cells are known to be cleared within 21 days of transplantation [6].

## 5. Conclusions

This TE cardiac graft seeded with hiPSC-CMs and human dermal fibroblasts improved LV systolic and diastolic functions on invasive hemodynamic assessment. Our data illustrate the advantages of using a TE scaffold to deliver hiPSC-CMs to treat CHF as opposed to direct cell injection into the heart or down the coronary arteries. The TE graft enables the cells to remain in contact with the epicardial surface of the heart, which has been shown to be a hub for cardiac regeneration. Another advantage of a TE graft is the ability to place the graft directly on the scar tissue. This is contrasted with direct cell injection, where it is not always clear where the cells are injected, and the cells may be rapidly washed out of the heart. These improvements in cardiac function occurred in the presence of a detectable immune response against the graft, specifically an increasing donor-specific antibody titer that peaked 21 days post-implantation of the xenograft. *In vitro* assessment of the graft revealed an electrophysiologic and mechanical phenotype consistent with hiPSC-CMs, but a complex messenger RNA profile suggesting rich soluble factor secretion.

## Data Availability

All data supporting the conclusions of this study are contained within the manuscript or within the manuscript's supplemental materials.

## Supplementary Materials

Supplementary Materials Supplemental Figure 1: surgical deployment of graft. Photographs of the engineered cardiac graft immediately prior to surgical implantation. The graft is observed (A) in the transport petri dish as the surgeon prepares for manipulation, (B) as the surgeon is actively handling the graft for surgical implantation, and (C) after the first suture has been placed to secure the engineered cardiac graft to the rat's epicardium. Supplemental Figure 2: rat invasive hemodynamics. A bar graph presenting invasive hemodynamic data as mean ± standard error of the mean. Sham (*n* = 17) data was collected at 6 weeks, while CHF (*n* = 17) and graft therapy (*n* =11) data were collected at 10 weeks. CHF PDP contains *n* = 15. Abbreviations are defined as follows: EDP: left ventricular end-diastolic pressure; SP: left ventricular systolic pressure; ±*dP*/*dt*: change in the left ventricular pressure over change in time; PDP: left ventricular peak-developed pressure. ^∗^ANOVA-Tukey versus sham, *p* < 0.05. ^#^ANOVA-Tukey versus CHF, *p* < 0.05. Supplemental Figure 3: rat echocardiography. A bar graph presenting transthoracic echocardiographic data (short-axis M mode) as mean ± standard error of the mean. Sham (*n* = 17) data was collected at 6 weeks, while CHF (*n* = 22) and graft therapy (*n* = 12) data were collected at 10 weeks. Abbreviations are defined as follows: EF: left ventricular ejection fraction; FS: left ventricular fractional shortening; Sys: systolic; Dia: diastolic; iDiam: left ventricular internal diameter; Volume: left ventricular volume; AW: left ventricular anterior wall thickness. ^∗^ANOVA-Tukey versus sham, *p* < 0.05. ^#^ANOVA-Tukey versus CHF, *p* < 0.05. Supplemental Figure 4: field potentials from engineered cardiac grafts. Field potentials obtained from the Maestro MEA (Axion BioSystems), each in its own individual well with 64 flat gold electrodes. (A) Sinus-like regular field potentials with negative or biphasic QRS complexes and clearly discernable T waves in nine independent engineered cardiac grafts. (B) Application of nonspecific beta-adrenergic receptor agonist isoproterenol revealed graded increases in beat rate (RR interval) and graded decreases in QT interval. (C) Application of nonspecific beta-adrenergic receptor antagonist sotalol revealed graded decreases in beat rate (RR interval) and graded increases in QT interval. Supplemental Figure 5: cycle length and calcium flux characteristics. Fura4 florescent calcium indicator and thapsigargin, an irreversible SERCA2a inhibitor, were utilized on engineered cardiac grafts (*n* = 5). Each engineered cardiac graft was assessed prior to exposure to thapsigargin so as to generate the control dataset. (A) Calcium (Ca^2+^) flux rise time in milliseconds (ms) versus cycle length in ms. The control grafts exhibited relative consistency in Ca^2+^ rise time (black circles) while thapsigargin-treated grafts (white circles) had a statistically significant delay in rise time at higher cycle lengths. ^∗^Two-tailed unpaired *t*-test versus the control, *p* < 0.05. (B) Ca^2+^ flux duration to fifty percent in ms versus cycle length in ms. No statistically significant relationships were found between the control (black circles) and thapsigargin-treated grafts (white circles). Supplemental Video 1: *in vitro* graft exhibits spontaneous and synchronous mechanical activity. Video of engineered cardiac graft in 6-well place (10x magnification). The synthetic bioabsorbable biomaterial of the graft can be visualized as woven bundles of fibers, creating a lattice extracellular matrix for the human dermal fibroblasts and human induced pluripotent stem cell-derived cardiomyocytes. Wave fronts of spontaneous and synchronous mechanical activity can be observed via diffraction of the microscope fluorescent light.
