# Supplementary material for: Epicardially Placed Bioengineered Cardiomyocyte Xenograft in Immune-Competent Rat Model of Heart Failure
Source: Stem Cells Int. 2021 Jul 24;2021:9935679. doi: 10.1155/2021/9935679 (PMC8325579; doi:10.1155/2021/9935679)
Supplement: Supplementary Materials — Supplemental Figure 1: surgical deployment of graft. Photographs of the engineered cardiac graft immediately prior to surgical implantation. The graft is observed (A) in the transport petri dish as the surgeon prepares for manipulation, (B) as the surgeon is actively handling the graft for surgical implantation, and (C) after the first suture has been placed to secure the engineered cardiac graft to the rat's epicardium. Supplemental Figure 2: rat invasive hemodynamics. A bar graph presenting invasive hemodynamic data as mean ± standard error of the mean. Sham (n = 17) data was collected at 6 weeks, while CHF (n = 17) and graft therapy (n =11) data were collected at 10 weeks. CHF PDP contains n = 15. Abbreviations are defined as follows: EDP: left ventricular end-diastolic pressure; SP: left ventricular systolic pressure; ±dP/dt: change in the left ventricular pressure over change in time; PDP: left ventricular peak-developed pressure. ∗ANOVA-Tukey versus sham, p < 0.05. #ANOVA-Tukey versus CHF, p < 0.05. Supplemental Figure 3: rat echocardiography. A bar graph presenting transthoracic echocardiographic data (short-axis M mode) as mean ± standard error of the mean. Sham (n = 17) data was collected at 6 weeks, while CHF (n = 22) and graft therapy (n = 12) data were collected at 10 weeks. Abbreviations are defined as follows: EF: left ventricular ejection fraction; FS: left ventricular fractional shortening; Sys: systolic; Dia: diastolic; iDiam: left ventricular internal diameter; Volume: left ventricular volume; AW: left ventricular anterior wall thickness. ∗ANOVA-Tukey versus sham, p < 0.05. #ANOVA-Tukey versus CHF, p < 0.05. Supplemental Figure 4: field potentials from engineered cardiac grafts. Field potentials obtained from the Maestro MEA (Axion BioSystems), each in its own individual well with 64 flat gold electrodes. (A) Sinus-like regular field potentials with negative or biphasic QRS complexes and clearly discernable T waves in nine independent engineered ca [file 9935679.f1.zip › 9935679.f1/iPSC Xenograft HF Supplemental Figure 1_PUBLISH.pdf]

Supplemental Figure 1: Surgical Deployment of Graft

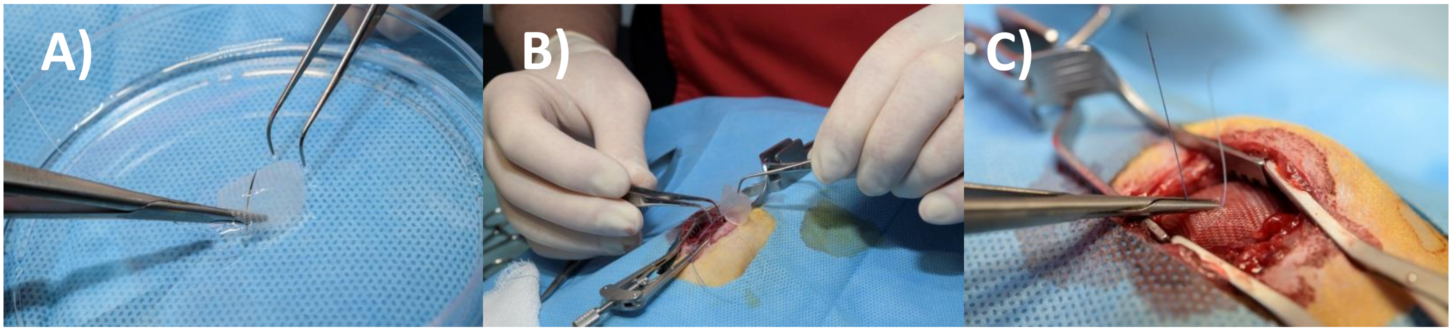

Supplemental Figure 1. Photographs of the engineered cardiac graft immediately prior to surgical implantation. The graft is observed A) in the transport petri dish as the surgeon prepares for manipulation, B) as the surgeon is actively handling the graft for surgical implantation, and C) after the first suture has been placed to secure the engineered cardiac graft to the rat's epicardium.
