# Supplementary material for: Epicardially Placed Bioengineered Cardiomyocyte Xenograft in Immune-Competent Rat Model of Heart Failure
Source: Stem Cells Int. 2021 Jul 24;2021:9935679. doi: 10.1155/2021/9935679 (PMC8325579; doi:10.1155/2021/9935679)
Supplement: Supplementary Materials — Supplemental Figure 1: surgical deployment of graft. Photographs of the engineered cardiac graft immediately prior to surgical implantation. The graft is observed (A) in the transport petri dish as the surgeon prepares for manipulation, (B) as the surgeon is actively handling the graft for surgical implantation, and (C) after the first suture has been placed to secure the engineered cardiac graft to the rat's epicardium. Supplemental Figure 2: rat invasive hemodynamics. A bar graph presenting invasive hemodynamic data as mean ± standard error of the mean. Sham (n = 17) data was collected at 6 weeks, while CHF (n = 17) and graft therapy (n =11) data were collected at 10 weeks. CHF PDP contains n = 15. Abbreviations are defined as follows: EDP: left ventricular end-diastolic pressure; SP: left ventricular systolic pressure; ±dP/dt: change in the left ventricular pressure over change in time; PDP: left ventricular peak-developed pressure. ∗ANOVA-Tukey versus sham, p < 0.05. #ANOVA-Tukey versus CHF, p < 0.05. Supplemental Figure 3: rat echocardiography. A bar graph presenting transthoracic echocardiographic data (short-axis M mode) as mean ± standard error of the mean. Sham (n = 17) data was collected at 6 weeks, while CHF (n = 22) and graft therapy (n = 12) data were collected at 10 weeks. Abbreviations are defined as follows: EF: left ventricular ejection fraction; FS: left ventricular fractional shortening; Sys: systolic; Dia: diastolic; iDiam: left ventricular internal diameter; Volume: left ventricular volume; AW: left ventricular anterior wall thickness. ∗ANOVA-Tukey versus sham, p < 0.05. #ANOVA-Tukey versus CHF, p < 0.05. Supplemental Figure 4: field potentials from engineered cardiac grafts. Field potentials obtained from the Maestro MEA (Axion BioSystems), each in its own individual well with 64 flat gold electrodes. (A) Sinus-like regular field potentials with negative or biphasic QRS complexes and clearly discernable T waves in nine independent engineered ca [file 9935679.f1.zip › 9935679.f1/iPSC Xenograft HF Supplemental Materials_PUBLISH.docx]

*Title:*

Epicardial Cardiomyocyte Xenograft May Improve Cardiac Function in Immune-competent Rodent Model of Heart Failure

*Running Head:*

Bioengineered Xenograft Cell Therapy for Heart Failure

*Authors & Affiliations:*

Ikeotunye Royal Chinyere PhD ^a^, Pierce Bradley MD ^a^, Joshua Uhlorn BS ^b^, Joshua Eason MD ^a^, Saffie Mohran BS ^c^, Giuliana G Repetti MD ^a^, Sherry Daugherty BS ^a^, Jen Watson Koevary PhD ^c^, Steven Goldman MD ^a^, Jordan J Lancaster PhD ^a^

1. Sarver Heart Center, University of Arizona, Tucson, AZ
2. Physiological Sciences GIDP, University of Arizona, Tucson, AZ
3. Department of Biomedical Engineering, University of Arizona, Tucson, AZ

**Manuscript Supplemental Materials**

**Supplemental Methods**

*Induced Pluripotent Stem Cell-derived Cardiomyocytes*

A novel line of hiPSC-CMs was provided *in-kind* by Fujifilm Cellular Dynamics International (Madison, WI, USA). Cells were aliquoted into suspensions, frozen, mailed overnight on dry ice, and thawed upon arrival before resuspension in RPMI 1640 media with B27 supplement.

Resuspended hiPSC-CMs were integrated into engineered grafts and quantitative polymerase chain reaction was utilized to evaluate the relative abundance of several amplicons. Specifically, the amplicons connexin 43 (*CXN43*), kinase insert domain receptor (*KDR*), smooth muscle actin (*SMA*), sarcomeric alpha actinin (*SAA*), cluster of differentiation 56 (*CD56*), and platelet-derived growth factor receptor-α (*PDGFRA*). Messenger ribonucleic acid expression levels from engineered grafts were normalized to expression levels from fibroblast-only cardiac grafts that did not contain hiPSC-CMs.

*Human Dermal Fibroblasts*

Human neonatal dermal fibroblasts were isolated from fresh healthy foreskin (Avery Therapeutics Inc., Tucson, AZ, USA). Isolated fibroblasts were stored at -196°C until thawing, and were then plated in 10% fetal bovine serum in DMEM (Gibco, Life Technologies; Grand Island, NY, USA) supplemented with Penicillin Streptomycin (100x), Antibiotic-Antimycotic (100x), and bicarbonate.

Cultures were maintained at 37°C and 5% carbon dioxide with media changes every 48 hours. Once cultures approached 85% confluence, fibroblasts were disassociated from the culture plates using 0.25% Trypsin, centrifuged, and resuspended in fibroblast-specific culture medium at 2x10^6^ cells per milliliter in preparation for integration into the bioabsorbable scaffold.

*Graft Preparation*

A bioabsorbable polyglactin-910 mesh (Ethicon-Johnson&Johnson, Somerville, NJ, USA) was trimmed and sterilized to generate 1.6 centimeter-diameter constructs. Meshes were rinsed in 1X phosphate-buffered saline and cellularized according to previously published methods ^[6]^. The bioabsorbable mesh is completely resorbed between three and four weeks post-implantation via hydrolytic processes ^[8]^.

In brief, engineered grafts were cultured for 30 days (37°C and 5% CO_2_) in low-adhesion plates to facilitate hiPSC-CM and fibroblast integration. Engineered graft confluence was achieved with 1:1 to 1:2 ratios of fibroblasts:hiPSC-CMs [6]. Culture media was changed every 24 hours until *in vitro* evaluation or surgical implantation of the graft onto the rat epicardium.

*Myocardial Infarction, Chronic Heart Failure, and Graft Deployment*

Immune-competent adult male Sprague-Dawley rats (Envigo, Indianapolis, IN, USA) 6-8 weeks of age were enrolled in this study under the guidance of Institutional Animal Care and Use Committee-approved protocols at the University of Arizona Animal Care Program and in compliance with the National Institute of Health’s ‘Guide for the Care and Use of Laboratory Animals’. As previously described, CHF was induced by left coronary artery ligation and the rats were recovered for 3 weeks before initiating treatment.

Rats underwent oropharyngeal intubation and ventilation (Harvard Apparatus, Holliston, MA, USA) and were induced using 3% volatile isoflurane in 100% oxygen before receiving an intraperitoneal saline-based cocktail of Ketamine (50mg/kg) + Xylazine (5mg/kg) + Acepromazine (1mg/kg) +Atropine (0.5mg/kg). A left thoracotomy exposed the heart through the chest wall so that a 5-0 TiCron (Covidien, Minneapolis, MN, USA) ligature was fastened proximally in the vicinity of the left coronary artery. The heart was returned to the chest as epicardial lidocaine and epinephrine were applied. Successful ligation was visually confirmed via immediate blanching of the myocardium in the circulatory territory. The chest muscle was sutured in layers using 2-0 silk purse string suture (with maximal lung inflation to remove air) and surgical staples were used to close the skin.

The rats were transferred from the heated surgical table to a warmed surgical pad and maintained on the ventilator until conscious. For post-operative pain, rats were provided intraperitoneal Buprenorphine SR (0.8 mg/kg; Wildlife Pharmaceuticals, Windsor, CO, USA) and maintained on Carprofen (5mg/kg) for up to seventy-two hours during daily monitoring. SHAM-operated rats underwent the surgical approach without coronary artery ligation and were maintained for six weeks.

*Echocardiography*

Rats were anesthetized with 1.5% isoflurane in 100% oxygen and laid supine on a warming pad with dorsal paw electrodes. Transthoracic echocardiography was performed with a dedicated rodent echocardiography system (Vevo2100 with 13-25MHz linear transducer, FUJIFILM VisualSonics, Toronto, ON, Canada) by an operator blinded to the treatment group within three days of the terminal study (10 weeks post-myocardial infarction for CHF rats and 6 weeks for SHAM rats) with views through the parasternal short and long axis as well as two-chamber apical views to evaluate the anterior, lateral, antero-lateral, inferior and posterior walls.

*Left Ventricular Hemodynamics*

Invasive hemodynamic data were obtained during the terminal study. Rats were anesthetized with intraperitoneal Inactin (100 mg/kg; Sigma-Aldrich, St. Louis, MO, USA), placed on a heated operating table, intubated, and ventilated. A 3French solid-state pressure transducer (ADInstruments, Colorado Springs, CO, USA) was equilibrated then inserted via the right carotid artery, and advanced into the left ventricle (LV) by an operator blinded to the treatment group. Data was digitized at a rate of 1000 hertz after approximately fifteen minutes after catheter placement for hemodynamic stabilization.

*In Vivo Cardiac Electrophysiology*

After invasive hemodynamic analysis, rats underwent a cardiac electrophysiology (EP) study. In brief, a three-lead electrocardiogram was obtained (ADInstruments) while programmed electrical stimulation protocols were performed (MATLAB, Natick, MA, USA). Capture threshold was determined before S1-S2 drivetrains were initiated to induce sustained ventricular tachycardia. The longest S1-S2 interval in milliseconds (msec) that failed to consistently capture the heart was reported as the minimum value for the ventricular effective refractory period (ERP).

*Ex Vivo Diastolic Pressure-Volume Relationships*

At the conclusion of the terminal study, the heart was arrested in diastole with potassium chloride and removed from the mediastinum. The whole heart was weighed (Sartorius Basic Digital Scale – Mettler Toledo, Columbus OH, USA) before the [right ventricle](https://www.sciencedirect.com/topics/medicine-and-dentistry/right-ventricle) was excised to prevent passive loading on the left ventricle during the Langendorff preparation.

A double-lumen catheter, one lumen attached to a pressure transducer (Millar, Houston, TX, USA) and the other lumen attached to an infusion pump with saline (Harvard Apparatus), was tightly secured around the [aortic root](https://www.sciencedirect.com/topics/medicine-and-dentistry/aortic-root). Prior to activating the infusion pump, gentle [aspiration](https://www.sciencedirect.com/topics/medicine-and-dentistry/aspiration) of the LV cavity was performed to remove any residual blood and reduce the pressure to −5 mmHg. The left ventricle was filled at a rate of 1.0 mL/min from 0 mmHg to 30 mmHg, without saline leakage, and emptied for repeat filling.

The LV stiffness constants were calculated over the following range of pressures: overall stiffness constant k_0_ from 2.5 to 30 mmHg, k_1_ from 0 to 3 mmHg, k_2_ from 3 to 10 mmHg, and k_3_ from 10 to 20 mmHg. Each value was the calculated as the tangential slope between points in the pressure-volume curves.

After the conclusion of the *ex vivo* pressure-volume (P/V) analysis, the LV was thoroughly dried with absorbent surgical gauze and weighed along with the dried right ventricle.

*In Vitro Graft Assessment*

Electrophysiologic and contractile properties of engineered grafts were defined with *in vitro* assays. Spontaneous beat rate of grafts was quantified via light microscopy and the qualitative degree of mechanical displacement was noted.

In addition, a multi-electrode array (NeuroNexus Inc., Ann Arbor, MI, USA) was used to record unipolar voltage electrograms from grafts during intrinsic electrical activity, as well as during pharmacologic adrenergic modulation, specifically with 1x10^-2^ Molar isoproterenol (MilliporeSigma, St. Louis, MO,USA), a non-selective beta agonist, and 1x10^-4^ Molar timolol (MilliporeSigma), a non-selective beta antagonist. Engineered grafts were maintained in a controlled-environment system (TC-202A, Warner Instruments–Harvard Bioscience Inc., Hamden, CT, USA) during the electrophysiologic analysis with the multi-electrode array.

Engineered graft field potentials were also plotted using a Maestro PRO MEA system (Axion Biosystems, Atlanta, GA, USA) with a 12-well plate (Cytoview MEA 12), each with 64 gold flat electrodes per well in an 8x8 array configuration. The grafts were maintained in a closed-loop system with precise environmental control, heated at 37°C with 5% carbon dioxide. Grafts in the wells were exposed to adrenergic manipulation in the form of stimulation via isoproterenol (MilliporeSigma), and inhibition in the form of sotalol (MilliporeSigma). Dose-dependent effects (isoproterenol: 10x10^-9^ Molar, 100x10^-9^ Molar, and 1000x10^-9^ Molar solutions; sotalol: 10x10^-9^ Molar and 100x10^-9^ Molar solutions) on beat rate and QT interval were investigated.

*Data Analysis*

The operators obtaining the end-point data were blinded with respect to the treatment groups in all *in vivo* studies. Data are expressed as mean ± standard error of the mean with p<0.05 determining statistical significance. For the *in vitro* graft evaluations, differences between groups were determined by two-tailed unpaired t tests. For the *in vivo* physiologic measurements, differences between groups were determined by one-way [Analysis of Variance](https://www.sciencedirect.com/topics/medicine-and-dentistry/analysis-of-variance) and Tukey post hoc testing. For immunologic measurements and *ex vivo* diastolic pressure-volume analyses, differences between groups were determined by two-tailed unpaired t test. The incidence of ventricular tachycardia was compared using Fisher’s exact test.

**Supplemental Results**

*Left Ventricular Hemodynamics*

Compared to SHAM rats (n=17), CHF rats (n=17) had changes in LV pressures associated with decompensation (Supplemental Figure 2). End-diastolic pressure (EDP) increased to 26±2 versus 5±1, p<0.0001, systolic pressure (SP) decreased to 116±3 versus 140±3 mmHg, p=0.0002, peak-developed pressure (PDP) decreased to 124±4 versus 185±4 mmHg, p<0.0001, LV ±dP/dt decreased 4554±209 versus 7672±183 mmHg/sec, p<0.0001 and -2737±102 versus -7380±186 mmHg/sec, p<0.0001, and Tau increased 37.9±2.4 versus 18.5±0.4 milliseconds, p<0.0001. Heart rate decreased in CHF rats (242±4 versus 285±4 beats-per-minute, p<0.0001).

Graft-treated CHF rats (n=11) exhibited a lower LV EDP compared to untreated CHF rats (n=17) (14±3 versus 26±2 mmHg, respectively. p=0.0005), a higher PDP (154±11 versus 124±4 mmHg, respectively, p=0.0057), a higher LV ±dP/dt (5686±556 versus 4554±209 mmHg/sec, respectively, p=0.0372 and -3855±492 versus -2737±102 mmHg/sec, respectively, p=0.0122), and lower Tau (30.7±2.5 versus 37.9±2.4 msec, respectively, p=0.0410) (Supplemental Figure 2).

*Echocardiography*

Compared to SHAM rats (n=17), CHF rats (n=22) had impaired LV function and an increase in systolic and diastolic volumes consistent with adverse LV remodeling (Supplemental Figure 3). Ejection fraction (EF) decreased (29±3 versus 69±3 %, p<0.0001) along with fractional shortening (FS) (15±2 versus 41±2 %, p<0.0001). LV systolic internal diameter (LVID) increased (9.2±0.3 versus 4.7±0.2 mm, p<0.0001) and diastole (10.8±0.2 versus 8.0±0.1 mm, p<0.0001). LV volume (Vol) in CHF also increased in systole (488±33 versus 109±11 µL, p<0.0001) and diastole (671±29 versus 344±12 µL, p<0.0001). Finally, LV anterior wall thickness (AW) decreased both in systole (1.5±0.1 versus 3.0±0.1 mm, p<0.0001) and diastole (1.3±0.1 versus 1.8±0.1 mm, p=0.0019).

Graft-treated rats (n=12) exhibited greater LV anterior wall thickness in systole versus CHF (2.1±0.1 versus 1.5±0.1 mm, respectively, p=0.0008, n=22). Graft-treated rats showed increased anterior wall thickness in diastole and nearly statistically significant improvements in echocardiography-derived cavity volume and internal diameter, consistent with the histopathologic findings of increased viable cardiomyocyte density in the anterior wall and decreased cavity diameter (Supplemental Figure 3).

*In Vivo Cardiac Electrophysiology*

In response to programmed electrical stimulation SHAM rats (n=9) exhibited no inducible ventricular tachycardia (VT) (0/9, 0%) and a short ERP (53±4 msec) (Figure 7). CHF rats (n=20) exhibited a higher incidence of inducible VT (12/20, 60%) compared to SHAM (p=0.0033) and a longer ERP (70±4) compared to SHAM (p=0.0314). Graft-treated rats showed no difference in the incidence of inducible VT (1/4, 25%) compared to SHAM (p=0.3002) and no change in ventricular ERP (81±21) compared to SHAM (p=0.5931).

*Ex Vivo Diastolic Pressure-Volume Relationships*

Analysis of stiffness constant k_1_ revealed that the initial phase of passive filling in CHF was altered compared to SHAM (13.2±1.0 versus 21.0±2.1 mmHg·g/ml, respectively, p=0.0087). This decrease in k_1_ for CHF was unchanged with graft therapy compared to untreated CHF (21.7±3.9 versus 13.2±1.0 mmHg·g/ml, p=0.1040). The calculated dead volume was greater for CHF than SHAM rats (0.46±0.05 versus 0.14±0.02 ml, p=0.0010) but was attenuated with graft-therapy compared to CHF (0.26±0.03 versus 0.46±0.05 ml, p=0.0045). The calculated operating LV end-diastolic volume for CHF also increased to 1.30±0.02 versus 0.39±0.02 ml in SHAM (p<0.0001) but was restored with graft therapy (0.74±0.03, p<0.0001). These changes in LV end-diastolic volume support reversal of maladaptive LV structural remodeling with the graft therapy.

**Supplemental Figure 1**: Surgical Deployment of Graft
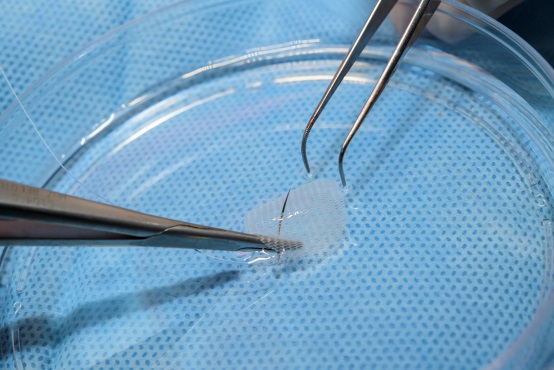

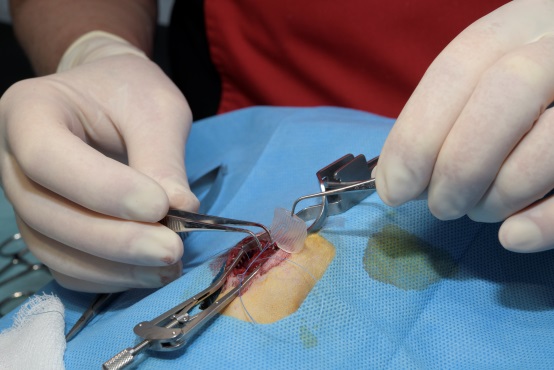

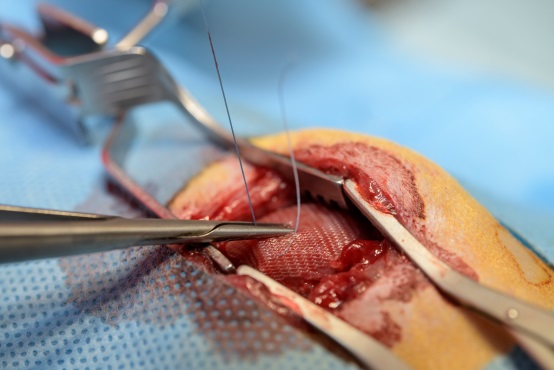


**A)**

**B)**

**C)**

**Supplemental Figure 1**. Photographs of the engineered cardiac graft immediately prior to surgical implantation. The graft is observed A) in the transport petri dish as the surgeon prepares for manipulation, B) as the surgeon is actively handling the graft for surgical implantation, and C) after the first suture has been placed to secure the engineered cardiac graft to the rat’s epicardium.

**Supplemental Figure 2:** Rat Invasive Hemodynamics


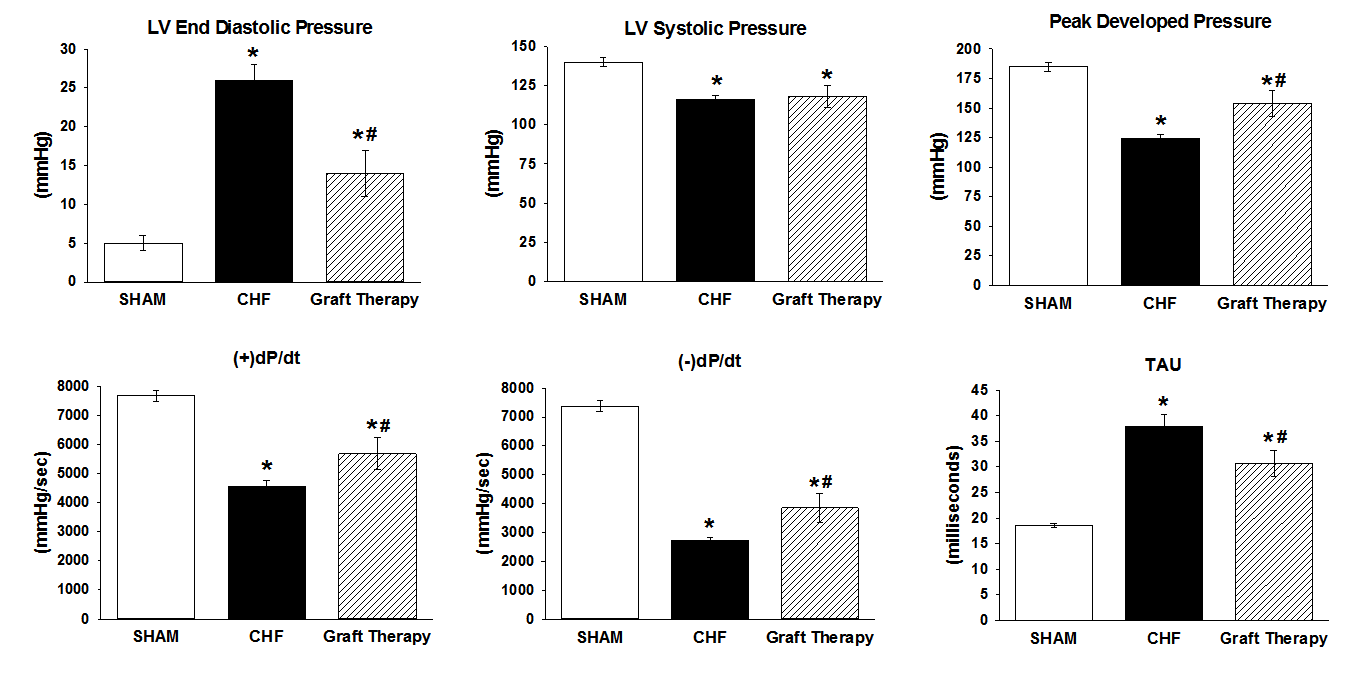


**Supplemental Figure 2**. A bar graph presenting invasive hemodynamic data as mean ± standard error of the mean. SHAM (*n*=17) data was collected at 6 weeks, while CHF (*n*=17) and Graft Therapy (*n*=11) data was collected at 10 weeks. CHF PDP contains *n*=15. Abbreviations are defined as follows: EDP: Left Ventricular End Diastolic Pressure, SP: Left Ventricular Systolic Pressure, ±dP/dt: change in Left Ventricular pressure over change in time, PDP: Left Ventricular Peak-Developed Pressure. ***** ANOVA-Tukey versus SHAM, p<0.05. **#** ANOVA-Tukey versus CHF, p<0.05.

**Supplemental Figure 3**: Rat Echocardiography


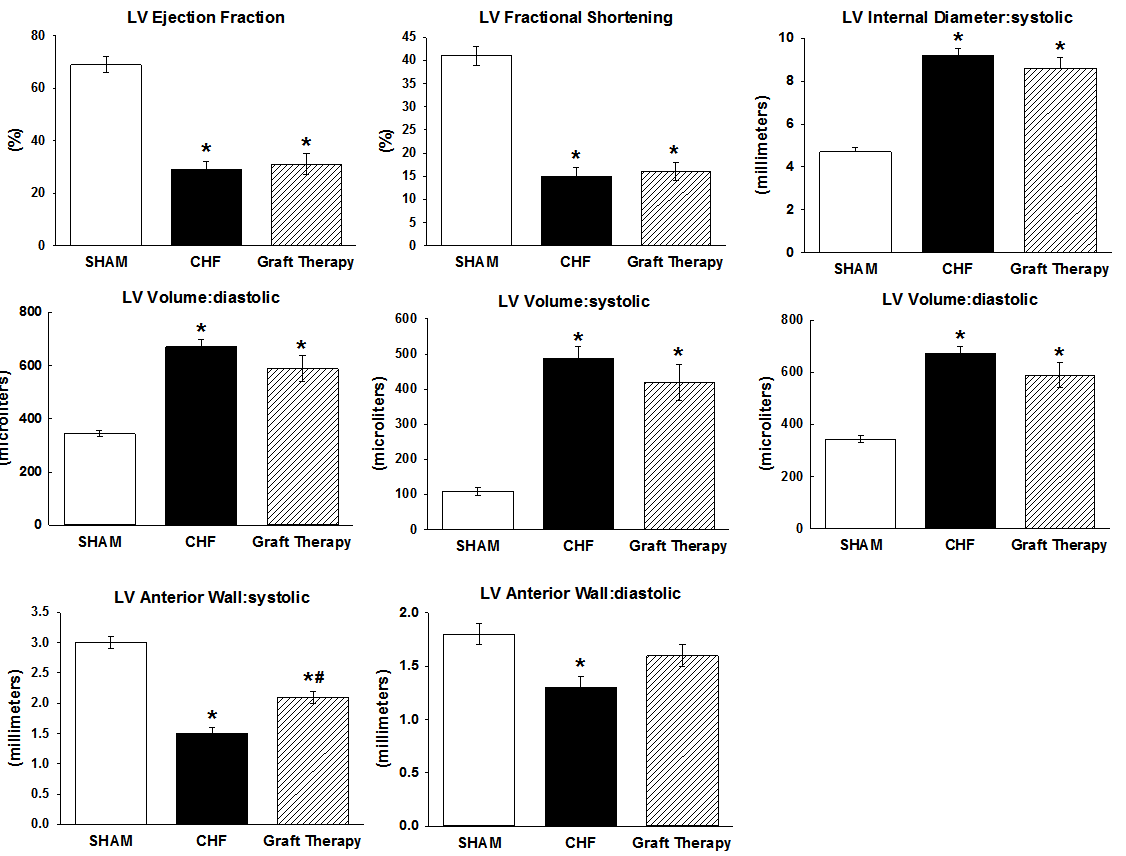


**Supplemental Figure 3**. A bar graph presenting transthoracic echocardiographic data (SAX M mode) as mean ± standard error of the mean. SHAM (*n*=17) data was collected at 6 weeks, while CHF (*n*=22) and Graft Therapy (*n*=12) data was collected at 10 weeks. Abbreviations are defined as follows: EF: Left Ventricular Ejection Fraction, FS: Left Ventricular Fractional Shortening, Sys: Systolic, Dia: Diastolic, iDiam = Left Ventricular Internal Diameter, Volume: Left Ventricular Volume, AW = Left Ventricular Anterior Wall thickness. ***** ANOVA-Tukey versus SHAM, p<0.05. **#** ANOVA-Tukey versus CHF, p<0.05.

**Supplemental Figure 4**: Field Potentials from Engineered Cardiac Grafts

**
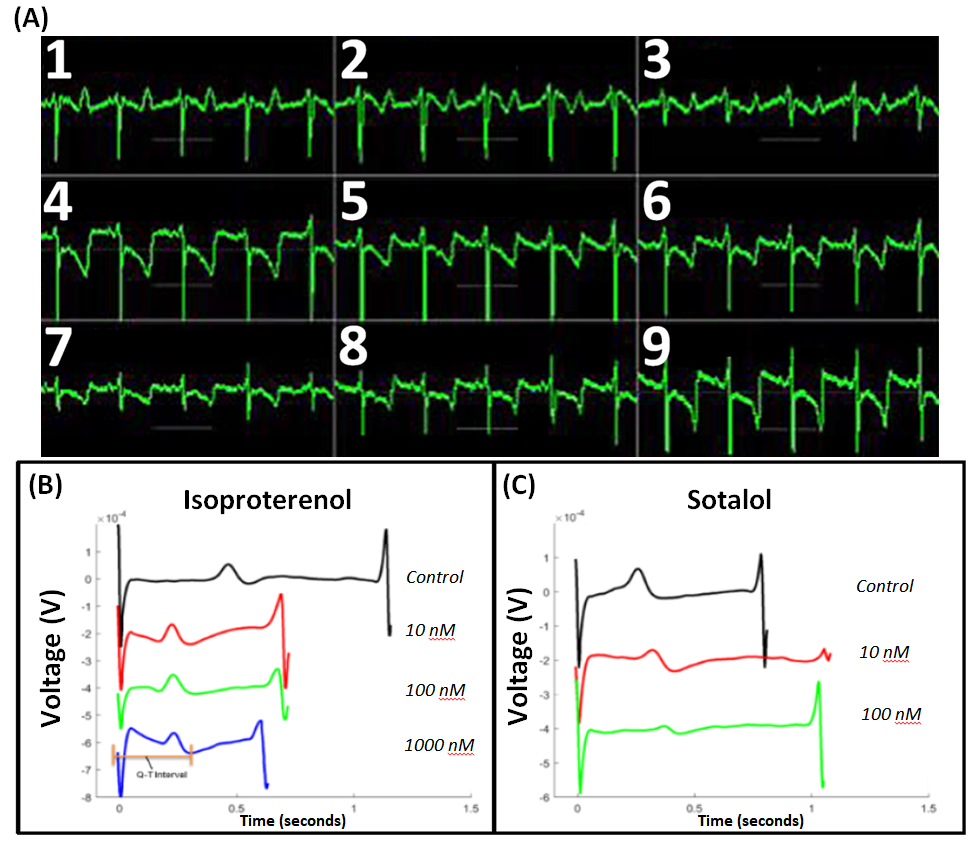
**

**Supplemental Figure 4.** Field potentials obtained from the Maestro MEA (Axion BioSystems), each in its own individual well with 64 flat gold electrodes.

**(**A) Sinus-like regular field potentials with negative or biphasic QRS complexes and clearly discernable T waves in nine independent engineered cardiac grafts.

(B) Application of non-specific beta-adrenergic receptor agonist Isoproterenol revealed graded increases in beat rate (RR interval) and graded decreases in QT interval.

(C) Application of non-specific beta-adrenergic receptor antagonist sotalol revealed graded decreases in beat rate (RR interval) and graded increases in QT interval.

**Supplemental Figure 5**: Cycle Length and Calcium Flux Characteristics

**
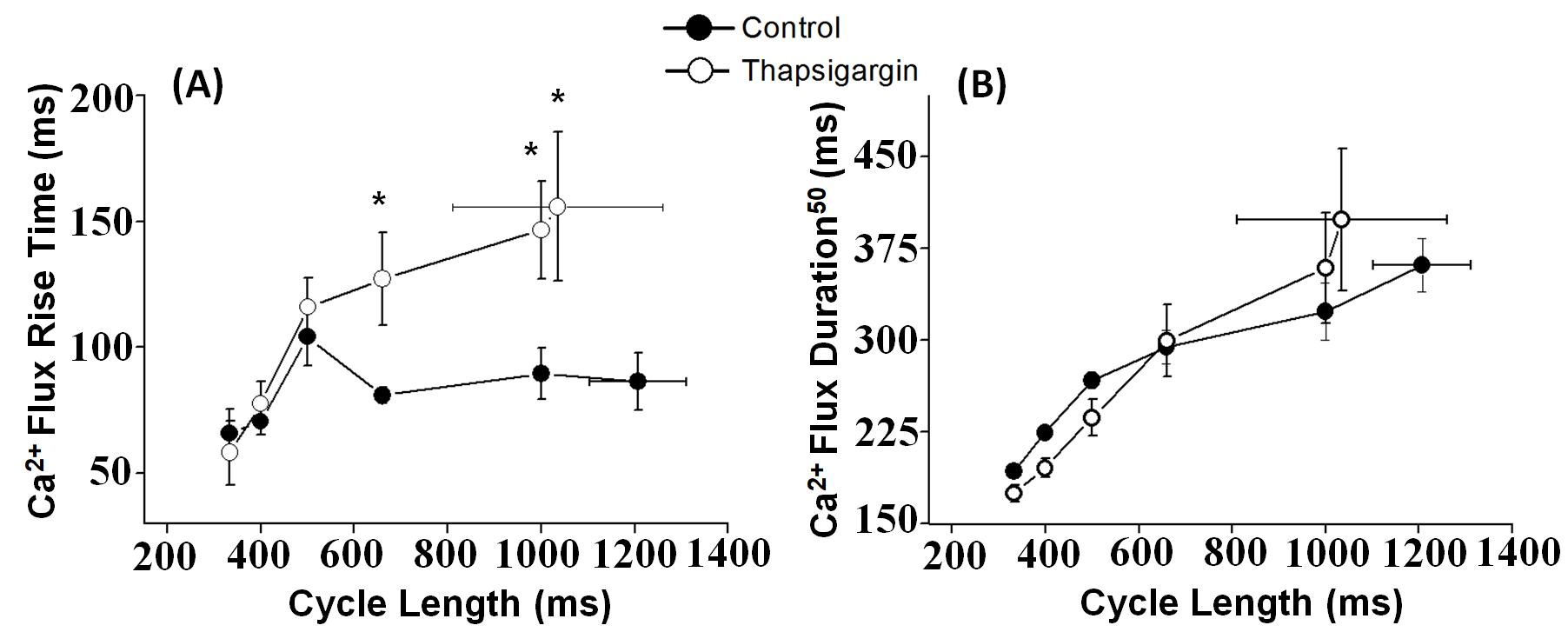
**

**Supplemental Figure 5.** Fura4 florescent calcium indicator and thapsigargin, an irreversible SERCA2a inhibitor, were utilized on engineered cardiac grafts (n=5). Each engineered cardiac graft was assessed prior to exposure to thapsigargin so as to generate the control dataset.

**(**A) Calcium (Ca^2+^) flux rise time in milliseconds (ms) versus cycle length in ms. The control grafts exhibited relative consistency in Ca^2+^ rise time (black circles) while thapsigargin-treated grafts (white circles) had a statistically significant delay in rise time at higher cycle lengths. * two-tailed unpaired t test versus Control, p<0.05.

(B) Ca^2+^ flux duration to fifty percent in ms versus cycle length in ms. No statistically significant relationships were found between the control (black circles) and thapsigargin-treated grafts (white circles).

**Supplemental Video 1**: *In Vitro* Graft Exhibits Spontaneous and Synchronous Mechanical Activity


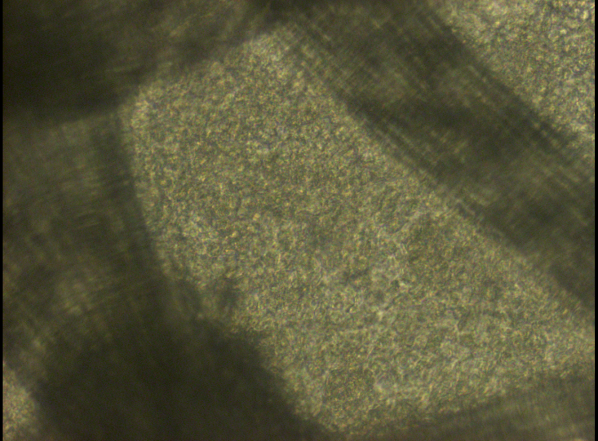


***Placeholder Still***

Double-Click Here →← Double-Click Here

**Supplemental Video 1**. Video of engineered cardiac graft in 6-well place (10X magnification). The synthetic bioabsorbable biomaterial of the graft can be visualized as woven bundles of fibers, creating a lattice extracellular matrix for the human dermal fibroblasts and human induced pluripotent stem cell-derived cardiomyocytes. Wave fronts of spontaneous and synchronous mechanical activity can be observed via diffraction of the microscope florescent light.
